# Erratum to “Optimal Hemodialysis Prescription: Do Children Need More Than a Urea Dialysis Dose?”

**DOI:** 10.4061/2011/702529

**Published:** 2011-11-28

**Authors:** Michel Fischbach, Ariane Zaloszyc, Betti Schaefer, Claus Peter Schmitt

**Affiliations:** ^1^Nephrology Dialysis Transplantation Children's Unit, University Hospital Hautepierre, Avenue Molière, 67098 Strasbourg, France; ^2^Division of Pediatric Nephrology, Center for Pediatric and Adolescent Medicine, INF 430, 69120 Heidelberg, Germany

All the authors' names were reversed. We would like to correct the author's names to be as follows:

Michel Fischbach instead of Fischbach Michel,
Ariane Zaloszyc instead of Zaloszyc Ariane,
Betti Schaefer instead of Schaefer Betti,
Claus Peter Schmitt instead of Schmitt Claus Peter.

